# Effect of female sex hormones on the developmental cycle of *Chlamydia abortus* compared to a penicillin-induced model of persistent infection

**DOI:** 10.1186/s12917-019-2013-7

**Published:** 2019-07-24

**Authors:** D. Álvarez, M. R. Caro, A. J. Buendía, C. Schnee, N. Ortega, A. Murcia-Belmonte, J. Salinas

**Affiliations:** 1 Departamento de Sanidad Animal, Facultad de Veterinaria. Campus de Espinardo, Universidad de Murcia, 30100 Murcia, Spain; 2 Departamento de Anatomía y Anatomía Patológica Comparadas, Facultad de Veterinaria, Regional Campus of International Excellence ‘Campus Mare Nostrum, Universidad de Murcia, Murcia, Spain; 3 Friedrich-Loeffler-Institut, Institute of Molecular Pathogenesis, Jena, Germany

**Keywords:** *Chlamydia abortus*, Ovine enzootic abortion, Female sex hormones, Penicillin, Persistence

## Abstract

**Background:**

*Chlamydia abortus*, an obligate intracellular pathogen with an affinity for placenta, causes reproductive failure. In non-pregnant animals, an initial latent infection is established until the next gestation, when the microorganism is reactivated, causing abortion. The precise mechanisms that trigger the awakening of *C. abortus* are still unknown. Sexual hormones such as estradiol and progesterone have been shown to affect the outcome of infection in other species of the family *Chlamydiaceae,* while estrogens increase chlamydial infection, progesterone has the opposite effect. To try to establish whether there is a relationship between these events and the latency/ reactivation of *C. abortus* in the reproductive tract of small ruminants, ovine endometrial (LE) and trophoblastic (AH-1) cells were treated with estradiol or progesterone prior to their infection with *C. abortus*. The results are compared with those obtained for treatment with penicillin prior to infection, which is a well-established model for studying persistent infection in other chlamydial species. Cells were examined by transmission electron microscopy, and an mRNA expression analysis of 16 genes related to the chlamydial developmental cycle was made.

**Results:**

The changes observed in this study by the action of sex hormones seem to depend on the type of cell where the infection develops. In addition, while the changes are morphologically similar to those induced by treatment with penicillin, the patterns of gene expression are different. Gene expression patterns therefore, seem to depend on the persistence induced models of *C. abortus* used. Hormone treatments induced aberrant forms in infected endometrial cells but did not affect the chlamydial morphology in trophoblast cells. At the genetic level, hormones did not induce significant changes in the expression of the studied genes.

**Conclusions:**

The results suggest that penicillin induces a state of persistence in in vitro cultured *C. abortus* with characteristic morphological features and gene transcriptional patterns. However, the influence of hormones on the *C. abortus* developmental cycle is mediated by changes in the host cell environment. Furthermore, a persistent state in *C. abortus* cannot be characterised by a single profile of gene expression pattern, but may change depending on the model used to induce persistence.

## Background

*Chlamydia abortus* is a widely spread obligate intracellular bacterium. The bacterium was initially associated with reproductive problems in small ruminants suffereing abortions, stillbirths or the birth of weak neonates [1], hence the name of the infection caused by the bacterium - ovine enzootic abortion. *C. abortus* infection has also been associated with reproductive problems in large ruminants [2, 3], and the variety of species in which the pathogenic action of *C. abortus* has been demonstrated is very large, ranging from *Canidae* [4] to *Delphinidae* [5]. In addition, *C. abortus* is potentially a professional zoonosis [6]. A particularly interesting feature of ovine enzootic abortion is the establishment of latent infections, since the infection remains unapparent in the non-pregnant animal and only becomes evident during a subsequent pregnancy [7]. While latency of infection and its subsequent recrudescence to trigger the onset of placental changes at a given time are recognised as characteristic features of *C. abortus* infection, the underlying mechanisms that control this series of events are poorly understood [7].

The sexual hormones estradiol and progesterone not only control the reproductive cycle, but also influence the progression of several pathogens by means of multiple mechanisms, including changes in host gene transcription and direct interactions with pathogens through pathogen-expressed hormone receptors [8]. Within the family *Chlamydiaceae,* it has been described that the in vitro pre-exposure of endometrial human cells to estradiol enhances the attachment of *C. trachomatis* EB to cell membranes and the development of chlamydial inclusions [9], while treatment with a combination of estradiol and progesterone significantly decreases this attachment [8]. These observations agree with the results obtained by Guseva et al. (2003) [10], who observed that female swine cells collected from the reproductive tract in the estrogen-dominant phase of the oestrous cycle were more susceptible to *Chlamydia suis* infection than cells obtained in the progesterone-dominant phase. Finally, it has been shown that the recrudescence of *C. abortus* in late-term pregnancy in sheep coincides with the marked physiological change in the serum estradiol/progesterone ratio that takes place during the final stage of gestation [11].

Chlamydiae have a unique biphasic developmental cycle, in which they alternate between two morphological forms, the infectious but metabolically less-active elementary body (EB) and the dividing, intracellular reticular body (RB), with a transitional form called intermediate body (IB). However, under stressful growth conditions, imposed by immunological responses, nutrient deprivation or antibiotics, the developmental cycle is disrupted, resulting in the appearance of large, pleomorphic, non-dividing RB, known as aberrant bodies (AB) [12]. Previous research suggests that the latent state of chlamydiae in vivo may be linked to the persistence phenotype in vitro [13, 14], which can be defined as the “viable but non-infectious” state of the pathogen. The AB phenotype is generally recognized as a hallmark of in vitro persistence, as well as differential mRNA expression patterns of some genes involved in the regulation of the developmental cycle, cell membrane structures and energy metabolism [13]. However, despite the general morphological similarities, significant differences in growth and ultrastructural characteristics have also been reported among systems of induction of persistence (reviewed in [13]). Even more evident differences have been described in the gene expression patterns of the different models of persistent infection [14–17].

In the literature, previous data from morphological, ultrastructural, and cell biological in vitro studies have shown that *C. abortus* is capable of persistence [18–20]. However, although the publication of the complete genome sequence of *C. abortus* [21] opened up the possibility for a systematic study of the transcriptional response during persistence, to our knowledge, such molecular changes have not been investigated for this pathogen.

Recently, it was demonstrated that a *Chlamydia trachomatis* persistence phenotype can be induced by sex hormones in vitro. In particular, estradiol supplementation of infected cells was shown to produce significant changes in the gene expression profile (for example, the up-regulation of the *trpB* gene, a reliable general marker for chlamydial persistence) and it also induced the development of atypical inclusions containing AB. Progesterone exposure resulted in a general up-regulation of genes that encode elements of carbohydrate and amino acid metabolism pathways [22].

Taken together, these observations led us to hypothesize that estradiol and progesterone may play a role in the mechanisms that regulate entry into and exit from the latent state in *C. abortus*. The current study was carried out to investigate morphological and transcriptional changes induced by these hormones during the developmental cycle compared to a penicillin-induced model of persistence used for the first time in *C. abortus*. Experiments were performed in vitro, using two cell lines obtained from ovine reproduction-related tissues as substrate for chlamydial growth.

## Results

### Morphological analysis

**Fig. 1:**
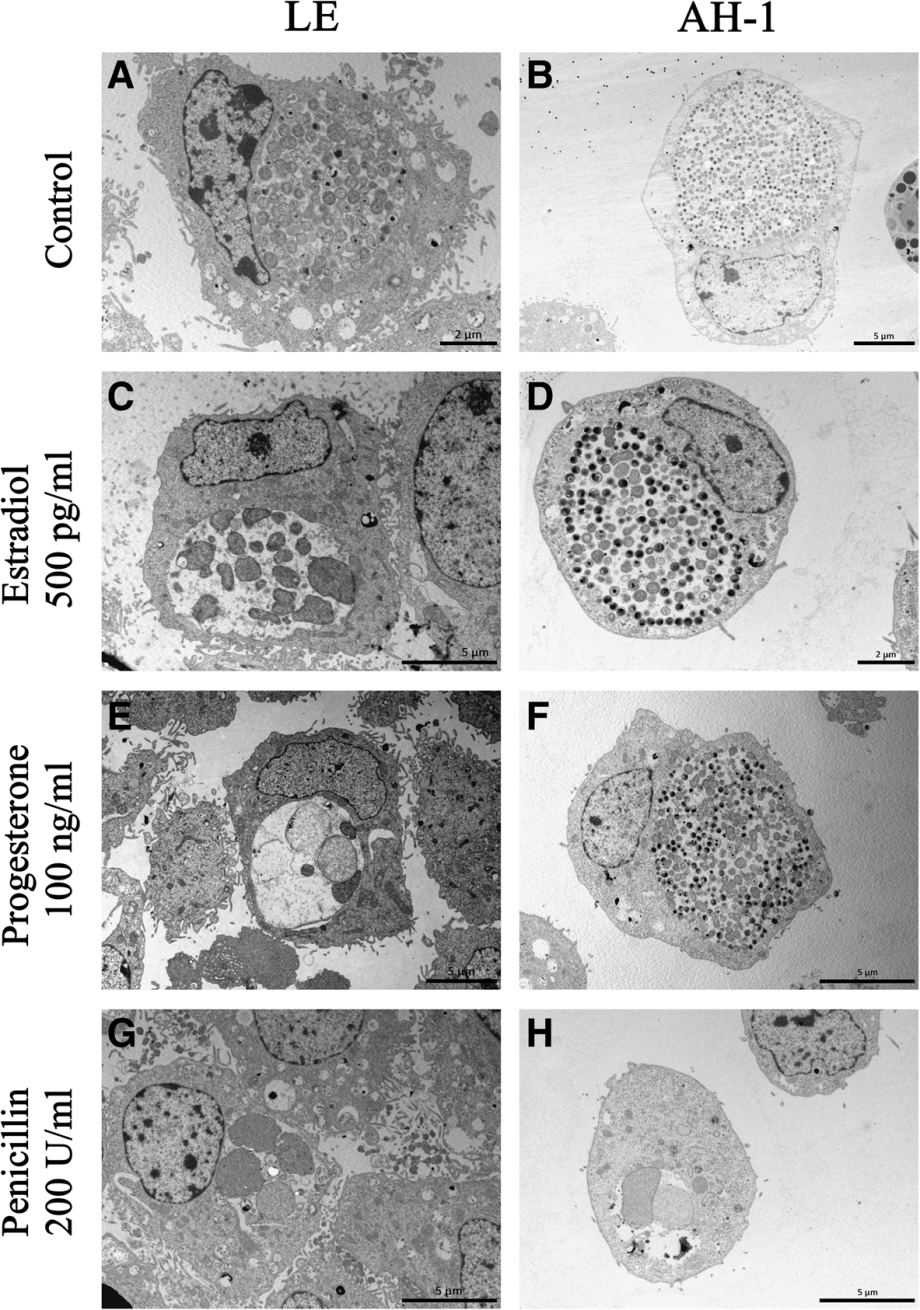
Transmission electron micrographs of *C. abortus* inclusions in LE (pictures in left column) and AH-1 cells (right column) at 72 h pi without treatment (**a**, **b**) or under estradiol (**c**, **d**), progesterone (**e**, **f**) or penicillin (**g**, **h**) treatment. **a** Untreated LE cell, normal inclusion containing numerous RBs and few IBs. **b** Untreated AH-1 cell showing a conventional large inclusion with RBs, IBs and EBs. **c** Pre-incubation with estradiol induced the presence of several pleomorphic enlarged RBs of different sizes and empty vesicle membranes within the inclusion in LE cells. **d** AH-1 cell treated with estradiol showing a typical mature inclusion with predominance of EBs and IBs over RBs. **e** Progesterone treated LE cell containing an inclusion with 2–4 abnormally enlarged atypical AB forms. **f** Progesterone supplemented AH-1 cell, large inclusion containing many RBs, IBs and EBs with normal shape and morphology. **g**, **h** Penicillin treated cultures showing characteristics consistent with a persistent chlamydial infection, both cell lines presented small inclusions with 2–4 giant ABs exclusively

**Fig. 2:**
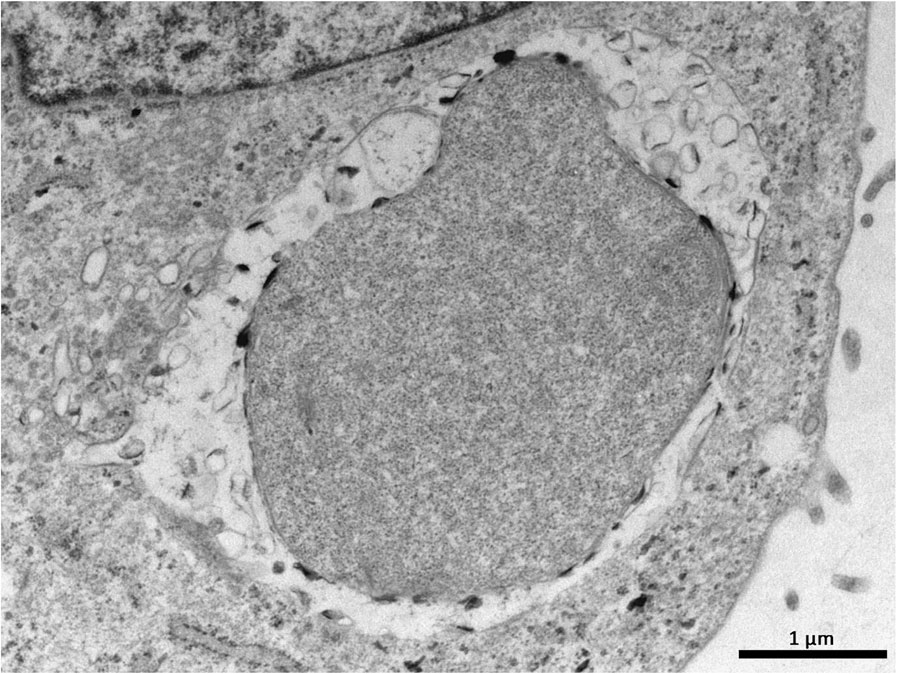
Transmission electron micrograph of a *C. abortus* inclusion in an infected AH-1 cell treated with 200 U/ml penicillin. Small inclusion containing a single large amorphous aberrant body. Multifocal deposition of electron-dense material can be observed on its outer membrane. Empty vesicle membranes from smaller reticular bodies are also present in the limited space of the vacuole left by the aberrant form

We compared the morphology of *C. abortus* inclusions in both LE and AH-1 cell cultures exposed (or not) to estradiol, progesterone or penicillin using transmission electron microcopy (TEM) (Fig. 1). Chlamydial inclusions that developed under regular culture conditions (e.g.. cell culture medium supplemented with charcoal stripped foetal bovine serum, without hormones nor penicillin) contained morphologically normal RBs in both LE and AH-1 cell lines (Fig. 1 a and b). In contrast, images of penicillin-treated cultures exhibited, in all infected cells, small inclusions containing 1–4 amorphous particles of up to 3 μm in length. Multifocal deposition of electron-dense material was observed on the outer membrane of these aberrant bodies, which sometimes shared the inclusion space with empty vesicle membranes from smaller RBs (Fig. 2). These findings were essentially identical in AH-1 and LE cells (Fig. 1g and h).

On the other hand, the supplementation of culture media with hormones led to differences in chlamydial morphology that depended on the host cell line used. Exposure of LE cells to estradiol induced the presence of sparsely populated inclusions containing aberrant forms, although to a lesser extent than in the case of treatment with penicillin. These abnormal RBs were very variable in number, size and shape, so that, within the same sample, it was possible to observe different inclusion types, ranging from normal (43% of all inclusions observed) to persistence appearance (32%), with intermediate morphologies (25%) also evident. (Fig. 1c). By contrast, morphological examination of estradiol-exposed AH-1 cells did not show any evidence of aberrant, persistent forms, but revealed typical chlamydial inclusion development, as represented by a mixture of characteristic EBs and RBs of normal size and shape (Fig. 1d). Likewise, when *C. abortus* was grown in progesterone-treated LE cells, TEM observation revealed enlarged pleomorphic RBs (Fig. 1e), similar to the aberrant forms described in penicillin-treated samples. This aberrant or persistent morphology prevailed among the observed inclusions (67%), although others of normal (20%) and intermediate (13%) morphology were also detected. On the other hand, when we applied the progesterone treatment to infected AH-1 cells, large normal inclusions containing many RBs, IBs and EBs of normal morphology could be seen (Fig. 1f).

### Gene expression

AH-1 and LE cells exposed to estradiol, progesterone or penicillin were infected with *C. abortus* AB7. Total mRNA was extracted at 48 and 72 h pi and reverse transcribed into cDNA. The relative expression of chlamydial genes coding for proteins involved in the stress response, membrane structure, cell division, regulation of RB-to-EB conversion and energy metabolism was investigated by q-PCR, using untreated controls as reference samples and the *16S rRNA* as endogenous control for normalization.

The relative expression of genes is summarized in Table 1. In general, treatment with penicillin caused a consistent up-regulation of genes related to the stress response and a down-regulation of genes related to membrane protein synthesis and cell division. In particular, chlamydia cultured in penicillin-treated LE cells showed a differential regulation at 48 h pi of 11 genes, of which 4 were up-regulated (*GroEL*, *dnaK*, *htrA* and *Cpaf*) and 7 down-regulated (*omcB*, *hctA*, *sctN*, *cydA*, *cydB* and *miaA*), while the same conditions in AH-1 cells led to differential expression of 5 genes, including 2 up-regulated (*dnaK* and *grpE*) and 3 down-regulated (*omcA*, *omcB* and *hctA*). At 72 h pi, the number of genes that were differentially expressed dropped to 8 in LE cells. In contrast, in AH-1 cells, at 72 h pi, the number of differentially regulated genes increased from 5 to 10 (*htrA*, *ompA*, *omcA*, *omcB*, *pmp17G*, *hctA*, *sctN*, *cydA*, *cydB*, *miaA*), all of them being down-regulated or shut down.

**Table 1:**
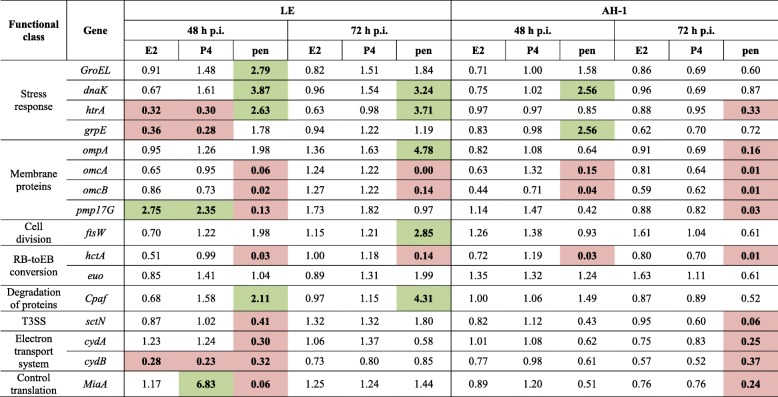
Relative mRNA expression levels (fold change (2^-ΔΔCt^)) of *C. abortus* genes at 48 and 72 h post infection in LE and AH-1 cells treated with estradiol (E2), progesterone (P4) or penicillin (pen). Data are normalized for the *16S rRNA* gene. Highlighted in green: significant up-regulation (2-fold cut-off); highlighted in red: significant down-regulation (cut-off < 0.5). T3SS: Type III secretion system

On the other hand, the gene expression profile analysis showed minor changes after treatment with female sex hormones, under our experimental conditions. The data show that only the expression of 4 genes in LE cells at 48 h pi was significantly altered by the addition of female sex hormones to the culture compared to infection in the absence of hormones. Out of these 4 genes, 3 were down-regulated, *htrA*, *grpE* and *cydB*, while only 1 was up-regulated, *pmp17G*. This differential expression was the same for both estradiol and progesterone treated cultures. Interestingly, no changes in the relative gene expression were detected in hormone treated AH-1 cells at any time studied post-infection, or in LE cells at 72 h pi.

## Discussion

A variety of studies have established that female reproductive hormones can influence the susceptibility and outcome of numerous infectious diseases in humans and animals [23, 24]. The fact that the reactivation and shedding of *C. abortus* is associated with certain times of the ovine reproductive cycle has been known for many years. Moreover, previous reports have shown how estradiol and/or progesterone influence the susceptibility of cells to infection with *C. trachomatis* [8, 9], *C. suis* [10] and *C. muridarum* [25]. It is, therefore, somewhat surprising that so little attention has been given to the role that reproductive hormones may play in the pathogenesis of OEA. The present work was designed to obtain data which will help to address this research gap.

The existing literature on chlamydial persistence is relatively extensive, but focuses mostly on human-infecting chlamydial species [13, 26], whereas, to our knowledge, only 3 in vitro models of persistence have been investigated for *C. abortus* to date: continuous culture [27], supplementation with IFN-γ [18, 19] and co-infection with porcine epidemic diarrhea virus [20]. In this study, an antibiotic-induced persistence system was assessed for the first time in *C. abortus* and it was used as a model to explore the relationship between female sex hormones and persistence in the pathogenesis of OEA.

β-lactams antibiotics such as penicillin kill bacteria through inhibiting peptidoglycan cross-linking activity, preventing new cell wall formation. In the current study, penicillin-stressed cultures showed altered growth and ultrastructural characteristics - small inclusions containing few severely enlarged aberrant RBs with the deposition of electron-dense material at their outer membrane and the presence of intrainclusional empty membranous material, similar to those described by Goellner et al. (2006) [15] in *C. psittaci*. The altered morphology of Chlamydial RBs reflected the changes expected in the expression of some of the genes studied. For instance, the membrane protein-encoding genes *omcA*, *omcB* and *pmp17G* and the histone like protein encoding gen *hctA*, were consistently down-regulated during the whole study, while others were up-regulated, such as *dnaK* and *htrA*, both transcribing for chlamydial stress response proteins. Together, these features suggest that penicillin blocked binary fission and RB-to-EB differentiation, is in line with observations obtained in studies with penicillin-stressed *C. trachomatis* [28], *C. pneumoniae* [29] and *C. psittaci* [15, 30]. This fact could have a significant impact on sheep farms since, even though penicillin is not the antimicrobial of choice against an outbreak of OEA, beta-lactams are the most frequently prescribed antibiotics to treat the main food-producing animals in Europe [31]. It is therefore likely that during the treatment of concurrent bacterial infections, developing chlamydiae in asymptomatic animals are exposed to beta-lactams, which, according to our results, would favour the entrance of *C. abortus* in a cryptic state, which, in turn, would contribute to the enzootic maintenance of the disease in the flock.

Many researchers have tried to find a common pattern of alterations in gene expression that correlates to the morphology of aberrant forms in vitro to characterize the persistent forms in vivo [12]. In the light of results obtained from penicillin-treated cultures, we suggest the following set of genes as markers to characterize the model of penicillin-induced persistence in *C. abortus*: *omcA* and *omcB* (membrane proteins), *dnaK* (stress response) and *hctA* (conversion of reticular bodies into elementary bodies). The expression of these 4 genes was consistently altered in both studied cell lines and at all moments post-infection.

On the other hand, it is noteworthy that hormones affect the chlamydial development differently, depending on the host cell type. Furthermore, the fact that aberrant forms were only seen in LE cells (maternal epithelial cells of the placentome) and not in trophoblastic cells, could be of interest for understanding the early mechanisms that underlie the pathogenesis of OEA. The placental infection by *C. abortus* starts at the maternal side of the placenta [32], and it is possible that high levels of progesterone during pregnancy contribute to maintain *C. abortus* in a latent state on the maternal side of the placenta. The sudden drop of progesterone levels at the end of pregnancy could be the signal that allows chlamydia to reactivate and invade the trophoblast, where the acute inflammatory response causes abortion.

Several studies have demonstrated that E2 and P4 modulate the innate immune responses of epithelial cells from the female reproductive tract [33, 34]. Wan et al. (2014) [35] analysed the transcriptome of human endometrial cells treated with E2 or P4 and infected with *C. trachomatis* and compared the quantity of chlamydial DNA between treated and untreated cultures by qPCR. The authors associated the lesser infectivity of P4-treated cultures with a significant up-regulation of genes related to multiple ways of the innate immunity in these cells. In order to explain the presence of aberrant forms in LE cells, further studies would be required to investigate the effect of hormones on the expression of mediators of the immune response during chlamydial infection. In the case of the AH-1 cell line, there are no studies about the effect of E2 and P4 on chlamydial growth in cells of trophoblastic origin. However, some cell responses to the infection have been reported. Thus, Wheelhouse et al. (2009) [36] determined that AH-1 cells produce TNF-α and CXCL8 in a dose- and time-dependent manner upon infection with *C. abortus*.

It is interesting to note that an aberrant morphology does not always correlate with the changes described in the expression of the genes studied, as seen in the P4 treated LE cells at 72 h pi. If we consider the development of aberrant forms as positive evidence of persistence, this finding means that the set of marker genes that are valid for characterizing the model of penicillin-induced persistence in *C. abortus* cannot be extrapolated to any other system of persistence. The idea that differences in transcriptional changes depend on the system of persistence induction has previously been suggested by other authors [12, 13, 15]. Furthermore, a previous study to analyse the effect of sex hormones on the transcriptome of *C. trachomatis* revealed that estradiol supplementation of infected cells induced significant changes in the expression of several chlamydial genes, including some that were also analysed in our study such as *omcB, cydA, cydB* and *miaA* [22]. Differences in the results obtained in the above mentioned study and ours suggest that changes in gene expression differ not only among systems of persistence induction, but also among different species of *Chlamydia*, even though morphological features of persistence are constant. This suggests that members of the *Chlamydiaceae* family have evolved more than one mechanism to attain the state of persistence and ensure their survival under adverse conditions. However, this study is a preliminary step, and more detailed host-pathogen RNA sequencing studies are needed to understand specific gene expression patterns in chlamydial persistence of *C. abortus*.

## Conclusions

In this study we have demonstrated that penicillin induces a persistent state in in vitro cultured *Chlamydia abortus*, with characteristic morphological features and gene transcriptional patterns. In addition, it is shown how female sex hormones may affect the chlamydial development through changes in the host cell environment. Although both penicillin and hormones induced the morphology of aberrant bodies under specific circumstances, the transcriptional response of the microorganism differed between treatments. This endorses the idea that chlamydial persistence cannot be defined by a common pattern of gene expression.

Our research provides new insights into the biology of the *C. abortus* persistent state. A better understanding of the relationship between the hormones that regulate reproductive cycles in ewes and the mechanisms that control the entry into and exit from the chlamydial persistent state would be useful for future applications such as the development of new vaccines or treatments that target chlamydia in the persistent state.

## Methods

### Host cell lines and culture

The experiments were carried out in two different cell lines derived from the ovine female genital tract and placenta: LE and AH-1.

The LE was an immortalized endometrial epithelial cell line isolated from the uterus of a sheep on day 5 of the oestrous cycle [37], and this cell line was a gift by the Center for Animal Biotechnology and Genomics (Texas A&M College of Veterinary Medicine, Texas, USA). Cells were grown in phenol red-free Dulbecco’s Modified Eagle Medium: Nutrient Mixture F-12 (DMEM/F12–1:1) supplemented with 10% Charcoal Stripped Fetal Bovine Serum, 2 mM L-glutamine, 10 μg/ml gentamicin and 2.5 μg/ml amphotericin B.

The AH-1 was an immortalized trophoblast cell line originally obtained from primary cultures of ovine placenta [38], and this cell line was a gift by the Department of Veterinary Microbiology and Pathology (Washington State University, Pullman, WA, USA). Cells were grown in phenol red-free Iscove’s Modified Dulbecco’s Medium (IMDM) supplemented with 10% Charcoal Stripped Foetal Bovine Serum, 2 mM L-glutamine, 10 μg/ml gentamicin and 2.5 μg/ml amphotericin B.

Both cell lines were maintained at 37 °C in humidified air containing 5% carbon dioxide. All cell culture reagents were purchased from Gibco.

### Hormone supplementation

Progesterone (P4) and 17β-estradiol (E2) (Sigma-Aldrich) were solubilised in absolute ethanol to make a stock solution, which was further diluted in phenol-free culture medium to attain the final concentrations. As serum levels of E2 and P4 fluctuate throughout the oestrous cycle in sheep [39], supraphysiological doses were used to cover the spectrum of possible special conditions such as local concentrations in uterus and pregnancy. Thus, sub-confluent cultures of AH-1 and LE cells were treated with E2 (500 pg/ml) or P4 (100 ng/ml) in 24-well plates (Falcon). Likewise, cells were treated with penicillin (200 U/ml) (Sigma-Aldrich) as stressor factor recognized to induce persistence in other *Chlamydiaceae* species. Untreated cells were used as negative control.

**Fig. 3:**
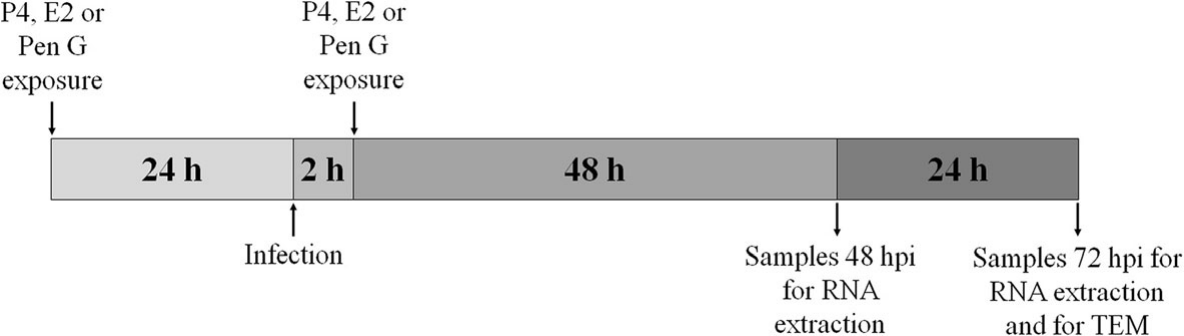
Experimental design. The diagram illustrates on a timeline the hormone or beta-lactam antibiotic exposure of AH1 and LE cells, infection and samples collection at different times. Cells were pre-exposed to progesterone (P4), estradiol (E2) or penicillin G (Pen G) added in incubation medium for 24 h. At this time, the cells were infected by the AB7 strain of *C. abortus* and, two hours later, they were again exposed to P4, E2 or Pen G in incubation medium, and incubated until 48 and 72 hpi, when samples were collected for genomic studies. In addition, samples were collected at 72 hpi for morphological studies using transmission electron microscopy (TEM)

Cell cultures were preincubated with hormones or penicillin 24 h before continuing with the experiments, and treatment exposure was continued throughout the study (Fig. 3).

### Microorganism and infection of cells

The *C. abortus* strain AB7 was grown in yolk sacs of developing chick embryos and titrated by counting inclusion-forming units (IFU) on McCoy cells according to a previously published protocol [40]. Standardised aliquots were frozen at − 80 °C until use.

Cell cultures were infected at an estimated multiplicity of infection (MOI) of 2. Briefly, chlamydial stock was diluted in a solution of diethylaminoethyl-dextran and phosphate buffered saline (DEAE-D/PBS, 1:10,000) to working concentration. After removal of the culture medium, 100 μl of chlamydial suspension was added to each well and plates were incubated at 37 °C for 1 h and 30 min. Then, they were centrifuged at 1400 X g for a further 30 min. Following infection, unbound EB were removed by aspiration of the suspension and fresh culture medium containing the respective hormone/penicillin treatments was added. Infected LE and AH-1 cells were incubated at 37 °C and in 5% CO_2_ for 48 and 72 h (Fig. 3).

### Morphological analysis

For transmission electron microscopy, cells were harvested 72 h post infection (h pi) and fixed with 2.5% glutaraldehyde in PBS for 1 h at 4 °C. Cell pellets were postfixed in 1% osmium tetroxide, dehydrated with increasing concentrations of absolute ethanol and embedded in epoxy resin using routine methods [41]. Areas of high cell density for ultrastructural examination were selected from epoxy resin blocks using semithin sections (1 mm) stained with Toluidine Blue. Ultrathin sections (80 nm) were mounted on copper grids, contrasted with uranyl acetate and lead citrate and examined with a transmission electron microscope (Philips Tecnai 12). Cells were classified into three types according to the morphology of chlamydial inclusions: normal, intermediate or persistent. The percentage of each type was calculated by counting and classifying cells in ten randomly selected fields in each sample.

### RNA extraction and reverse transcription

At 48 and 72 h pi total RNA was extracted from infected cells using a RNeasy Mini Kit (Qiagen) and DNA was removed from samples by DNase treatment, according to the instructions of the manufacturer. RNA concentrations were quantified using a Nanodrop ND-2000 spectrophotometer (Thermo Scientific). For each sample, 400 ng of total RNA was reverse transcribed into cDNA using random nonamers as primers and a Reverse Transcriptase Core kit (Eurogentec), following the manufacturer’s instructions.

### Gene selection and design of gene-specific primers

For this study, 16 genes of interest were selected based on a literature review of studies on gene expression during the chlamydial developmental cycle and persistence (Table 2). Since most reviewed articles were performed on the species *C. trachomatis* [22], *C. psittaci* [15] or *C. pneumoniae* [16], a multiple sequence alignment of the gene sequences with the *C. abortus* AB7 strain genome sequence available in GenBank (NCBI Reference Sequence: NZ_LN554882.1) was performed using the BLAST server from the National Center for Biotechnology Information (http://www.ncbi.nlm.nih.gov/blast/) to find the specific sequences of the genes of interest in the *C. abortus* genome.

**Table 2:** Gene targets and q-PCR primers used in this study with their derived proteins and functional classification. References include previous studies in which the target gene was investigated, chlamydial species studied and the model of persistence: PenG: penicillin; ID: iron depletion; IFN-γ, HS: Heat shock

Gene	Primer sequence (5′-3′)	Protein	Functional classification	Reference
*16S rRNA*	Fw: CTTGTACACACCGCCCGTC Rv: GCCCAACCTAGTCAAACCGTC	Ribosomal RNA	Reference gene	
*GroEL*	Fw: CAACAGGTAGCAGAATCCGGA Rv: CTCTTCGCTGATCAATTGGCCA	Heat shock protein 60	Stress response	Kalmar et al., 2015 [42] *C. abortus* and *C. psittaci*
*dnaK*	Fw: ACCAACCGTTCATCACTATGGA Rv: TGGCATGGAGCTTTTGTACG	Heat shock protein 70		Mukhopadhyay et al., 2006 [43] *C. pneumoniae*. ID, IFN-γ, HS.
*htrA*	Fw: CAAGATGACGGTGTCTCTGCTTT Rv: TGCTTCGACAGAAACAATCAGCA	Protease		Timms et al., 2009 [17] *C. pneumoniae*. IFN-γ, ID
*grpE*	Fw: CAACAGTTCCTACCCCCGATAA Rv: CCTCATCTGACATCTGTGAGGC	Cofactor Hsp70		Goellner et al., 2006 [15] *C. psittaci.* PenG, ID, IFN-γ
*ompA*	Fw: GCATTATTGTTTGCCGCTAC Rv: ATCACCTGAAGCACCTTCCCA	MOMP	Membrane proteins	Timms et al., 2009 [17] *C. pneumoniae*. IFN-γ, ID
*omcA*	Fw: TGCCGTATTGTAGATTGCTGCT Rv: TGAACTCCTGAATTGCACTCAG	outer mb protein A		Goellner et al., 2006 [15] *C. psittaci.* PenG, ID, IFN-γ
*omcB*	Fw: GTCGTATTCGATGCTCTGCCTA Rv: AGCAACGGGTACCGTTAAAGT	outer mb complex protein B		Amirshahi et al., 2011 [22] *C. trachomatis*. Estradiol and progesterone
*pmp17G*	Fw: GGGTGATTGGGGTAACGATTGT Rv: AGGTTGGTGAGATTGCTGCT	Polymorphic mb protein		Wheelhouse et al., 2009 [36] *C. abortus*
*ftsW*	Fw: TTGTTCCCTGCGTCGCTATC Rv: AAAAGCTATTACGGCTGCGGA	cell division mb protein	Cell division	Kalmar et al., 2015 [42] *C. abortus* and *C. psittaci*
*hctA*	Fw: ACGTGACTTAGACAAGGCCGA Rv: TGCTTTACGCTTAGATGCTGATT	DNA-binding histone-like protein	Regulation of RB-to-EB conversion	Goellner et al., 2006 [15] *C. psittaci.* PenG, ID, IFN-γ
*euo*	Fw: AAGGCTTCTAAAACAACCCGG Rv: GGCTAATAACCCAAGCAGCG	DNA binding protein		Goellner et al., 2006 [15] *C. psittaci.* PenG, ID, IFN-γ
*Cpaf*	Fw: CGCACCCTGAGCATCGTTA Rv: AAGACAAAACCCCCAGCTCCT	chlamydial protease-like activity factor	Degradation of proteins	Kalmar et al., 2015 [42] *C. abortus* and *C. psittaci*
*sctN*	Fw: TTCGATACCCTCATGTCGCAA Rv: TACCTCCCCAACGCGTACATT	type III secretion cytoplasmic ATPase	Type III secretion system	Goellner et al., 2006 [15] *C. psittaci.* PenG, ID, IFN-γ
*cydA*	Fw: GGCGTTTGCATCCAAGAGTTA Rv: GGTAGTGAAGGCGTTTTTGCTTT	cytochrome d ubiquinol oxidase sub I	Electron transport system	Amirshahi et al., 2011 [22] *C. trachomatis*. Estradiol and progesterone
*cydB*	Fw: TCCACACAACAACGTGTAGG Rv: TAGTAAGACCGAGTCAGCAAAATGG	cytochrome d ubiquinol oxidase sub II		Amirshahi et al., 2011 [22] *C. trachomatis*. Estradiol and progesterone
*miaA*	Fw: CGCCCACAAGAATAGGGACTT Rv: GACAGCGCATTCCTCATTATCTGA	isopentenylpyrophosphate transferase	Control at the translational level	Amirshahi et al., 2011 [22] *C. trachomatis*. Estradiol and progesterone

Primers were designed for each gene of interest using the tool OligoPerfect Designer (Thermo Scientific) and further evaluated with OligoAnalizer 3.1 (IDT). In accordance with the program’s instructions, melting temperatures (Tm) of primers were kept between 58 and 60 °C. Sequences of primers are presented in Table 2. All oligonucleotides were synthesized by Eurofins and tested at concentrations of 1, 0.5 and 0.25 μM (final concentration) by quantitative real-time PCR using genomic DNA of *C. abortus* as template.

### Quantitative real-time PCR analysis

Changes in gene expression were analysed using quantitative real-time polymerase chain reaction (q-PCR). For each real-time analysis, samples were run in triplicate, twice independently. All reactions were performed on an Applied Biosystems 7500 fast Real-Time PCR System (Thermo Scientific) using 10 ng of cDNA, 10 μl of SYBR Green Master Mix (Kapa SYBR Fast, Sigma-Aldrich) and 2 μl of each primer at 0.25 μM. Final reaction volumes were made up to a total volume of 20 μl with RNAse DNase-free H_2_O. The cycling conditions were 95 °C for 3 min, followed by 40 cycles of 95 °C for 3 s and 60 °C for 30 s, according to the Master Mix manufacturer’s instructions. A melting-curve analysis was performed to ensure specificity of the amplicons. Ct values of treated samples were related to those of untreated controls. The relative expression of the selected genes was normalized to the level of 16S rRNA gene in each sample and calculated using the comparative 2^-ΔΔCt^ method. To use this method, the efficiency of each PCR reaction was previously checked to be approximately 100%.

A 2-fold change in the normalized gene of interest expression level, which is a commonly established threshold in other chlamydial gene expression studies, was used as a cut-off [15, 22, 42, 43]. Thus, genes showing relative expression ratios higher than 2 or lower than 0.5 were considered to be up- or down-regulated respectively.

## Data Availability

All data supporting our findings are included in the manuscript. If readers need additional information and/or data sets, they will be provided by the corresponding author upon reasonable request.

## Abbreviations

AB: Aberrant form; EB: Elementary body
E2: 17β-estradiol
IB: Intermediate body
OEA: Ovine enzootic abortion
P4: Progesterone
PenG: Penicillin G
RB: Reticular body
